# Foraging on the wing for fish while migrating over changing landscapes: traveling behaviors vary with available aquatic habitat for Caspian terns

**DOI:** 10.1186/s40462-022-00307-8

**Published:** 2022-03-02

**Authors:** C. Rueda-Uribe, U. Lötberg, S. Åkesson

**Affiliations:** 1 Department of Biology, Centre for Animal Movement Research, Lund University, Ecology Building, 223 62 Lund, Sweden; 2 BirdLife Sweden, Stenhusa gård, Lilla Brunneby 106, 386 62 Mörbylånga, Sweden

**Keywords:** Fly-and-forage, Habitat selection, Migratory schedules, Optimal migration, Orientation and navigation, Route tortuosity, Tailwind, *Hydroprogne caspia*

## Abstract

**Background:**

Birds that forage while covering distance during migration should adjust traveling behaviors as the availability of foraging habitat changes. Particularly, the behavior of those species that depend on bodies of water to find food yet manage to migrate over changing landscapes may be limited by the substantial variation in feeding opportunities along the route.

**Methods:**

Using GPS tracking data, we studied how traveling behaviors vary with available foraging habitat during the long-distance migration of Caspian terns (*Hydroprogne caspia*), a bird with a specialized diet based on fish that needs bodies of water to forage. We measured individual variation in five traveling behaviors related to foraging along the route and used linear mixed effects models to test the following variables as predictors of traveling behaviors: proportion of overlap with water bodies, weather conditions, days at previous stopover and days of migration. Also, we tested if during traveling days flight height and speed varied with time of day and if birds were in areas with greater proportion of water bodies compared to what would be expected by chance from the landscape.

**Results:**

We found variation in migratory traveling behaviors that was mainly related to the proportion of overlap with water bodies and experienced tailwinds. Suggesting a mixed migratory strategy with fly-and-foraging, Caspian terns reduced travel speed, flew fewer hours of the day, had lower flight heights and increased diurnal over nocturnal migratory flight hours as the proportion of overlap with water bodies increased. Birds had lower flight speeds and higher flight heights during the day, were in foraging habitats with greater proportions of water than expected by chance but avoided foraging detours. Instead, route tortuosity was associated with lower wind support and cloudier skies.

**Conclusions:**

Our findings show how birds may adjust individual behavior as foraging habitat availability changes during migration and contribute to the growing knowledge on mixed migratory strategies of stopover use and fly-and-forage.

**Supplementary Information:**

The online version contains supplementary material available at 10.1186/s40462-022-00307-8.

## Background

The capacity of organisms to adjust to changing environmental conditions should have fitness benefits and be favored by selection [1]. In particular, successful animal migration should depend on how individuals adjust behaviors between and within seasons [2], because changing behaviors as weather and ecological variables vary along the route can reduce the costs of migration [3, 4] and even lead to the avoidance of hazardous weather events to ensure survival [5]. Although mechanistic explanations of decision-making remain unclear [6], research on birds has shown that individuals can select favorable winds [5, 7, 8], weather [9, 10], flight altitudes [11], routes [12, 13] and stopover frequency [14] and sites [15, 16]. Between years, changes in migratory timing for several species have also been documented [e.g. 12, 14, 17] and improvements in migratory performance have been evidenced as individuals age [18].

The importance of individuals adjusting migratory behaviors to environmental conditions is particularly evident in long-distance avian migrants that cross challenging landscapes such as deserts, oceans and mountains [19–21]. How migratory birds overcome ecological barriers has been studied for many species [e.g. 22–24], but changes in traveling behaviors in relation to foraging habitat structure and availability along the entire route is less understood. Foraging habitat availability is determinant in the opportunities birds have to fuel migration, and feeding habitat can be either abundant and evenly spaced or patchily distributed in the landscape, thus affecting optimal decisions of stopover use [25] or foraging behavior during traveling days [26].

For those migrants that forage while advancing along the route, available habitat determines feeding opportunities [26] and should alter the balance between the costs and benefits of a fly-and-forage strategy [27]. This type of migratory strategy may be advantageous for species that forage on the wing because they can continue covering distance as they find food, thus increasing their total speed of migration [27, 28]. A fly-and-forage migration could also have the benefits of reduced fuel loads during flight as a consequence of the continuous replacement of consumed energy. Possible costs arise with the time needed to find and handle food, lower traveling speeds and increased detours to find suitable foraging sites [27], and physiological challenges of switching between digestion and migratory flight that have not yet been addressed. This implies that whether a fly-and-forage strategy is favorable depends on the intrinsic biological factors of the species, as in its foraging habits, physiology and flight capabilities, but also on the environment. Considering that in long-distance migration ecological conditions will most probably vary along the route, optimal solutions for some migratory bird species should result in a mixed strategy of stopovers with fly-and-forage [27].

That may be the case of long-distance migrants that fly over large land masses but depend on bodies of water to forage. The behavior of such species may be particularly limited by the availability of foraging habitats along the route if they have a fly-and-forage strategy. An example that has been previously studied is the osprey (*Pandion haliaetus*), a species that feeds on fish and yet manages to forage on traveling days [27]. Klaassen et al. (2008) showed how their migratory behavior changed regionally between Europe and Africa, where there are stark differences in water availability due to the presence of the Sahara Desert. Ospreys had lower speeds, dedicated fewer hours to flight and were mostly found near bodies of water while migrating over Europe. Lower traveling speeds and less time for migratory flights are expected behavioral changes for fly-and-forage migrants because foraging necessarily requires finding, locating, catching, handling and digesting prey [26]. Other behaviors related to foraging during migration that may be quantified from position data of tracking devices include altered schedules that favor diurnal activity to visually detect foraging habitat and prey [27, 29], reduced time at stopovers [26], and route detours to explore and follow suitable foraging areas [30].

In this study, we used tracking data with high temporal resolution from GPS devices to analyze how migratory traveling behaviors vary with the availability of water bodies in the landscape and test if these behaviors are indicative of foraging during traveling days for Caspian terns (*Hydroprogne caspia*). Caspian terns breeding in the Baltic Sea are long-distance migrants [31–34] that have a specialized diet based on fish [35]. They are good fliers and forage with their heads directed downwards to visually detect their prey [36], making them possible fly-and-forage migrants. However, Caspian terns migrate an average of 7,111 km in autumn and 6,290 km in spring between their breeding grounds in the Baltic Sea and their wintering grounds in the Iberian Peninsula, Nile River Delta and sub-Saharan Africa [34]. Since their migration includes crossing continental Europe, and for some individuals also the Mediterranean Sea and Sahara Desert, behaviors related to a fly-and-forage strategy may be limited by the substantial variation in the availability of aquatic habitats along the route. Our specific research questions were: (1) Do the traveling behaviors of Caspian terns change during migration? (2) Is the availability of water bodies a major predictor of migratory traveling behavior for Caspian terns? (3) Do Caspian terns adjust flight speeds and heights according to the time of day while migrating? (4) Are terns in locations with more water than expected by chance from the surrounding landscape while advancing along their migratory route? We expected to find variation in travel speed, flight height above the ground, daily hours of flight, proportion of day and night time spent flying, and track tortuosity in relation to the drastic changes in landscape birds experience during migration. We predicted that if Caspian terns forage on traveling days, aquatic habitat availability should be a major predictor of traveling behavior. Where there is a greater proportion of water bodies along the route, Caspian terns should decrease travel speed, fly closer to the ground, shorten hours dedicated to migratory flight, use daytime hours for flight and take more detours. Also, a fly-and-forage migrant using vision to catch prey near the ground should fly at lower speeds and heights during daytime hours in comparison to the night. Finally, if the species is foraging while covering distance during migration, observed relocations should have greater proportions of water bodies than those expected by chance from the landscape.

## Methods

### Tracking and data processing

Complete migratory tracks were obtained from solar powered 15–20 g GPS-GSM/3G devices (Ornitela) mounted with Teflon leg harnesses on 39 Caspian terns (12 adults, 27 juveniles) from 5 breeding colonies in the Baltic Sea in Sweden, as explained in [34] and available in [37]. The tracks had a high temporal resolution (recordings occurred every 5 min to maximum 2 h depending on battery), and were processed to exclude low-quality points and resampled into 30-min intervals using the package *amt* in R [38]. Migratory tracks began when birds left either their breeding or wintering grounds for autumn and spring migration, respectively, and ended in the last “traveling” day before reaching the areas they occupied during the corresponding stationary months. Stopovers were defined as the areas where birds remained during days when they did not advance more than 35 km between last daily relocations [22, 30] because this threshold was over the average daily distance covered when birds were more sedentary (months January through March) and close to the median value separating the two modes when individual daily speeds deviated from an unimodal distribution (34.96 km/day, see [34]). Traveling segments were obtained from the tracks by excluding identified stopovers, so a traveling segment is the track of a bird when it is covering migration distance between stopover periods (see Additional file 1: Fig. S1 as an example and Additional file 1: Fig. S2 for all segments).

### Behavior in traveling segments

Five variables related to a fly-and-forage strategy were calculated from traveling segments to characterize migratory behavior: travel speed, flight height above the ground, daily hours of flight, day to night ratio and route tortuosity. Travel speed was calculated by dividing the sum of the travelled distance by the total number of days of the segment. We estimated flight altitude as recorded by GPS devices for relocations when birds were in flight in traveling segments (instantaneous ground speed greater than 10 km/h at both the start and end of 30-min intervals), and calculated flight height above the ground by subtracting ground elevation from flight altitude. Data on elevation for each relocation point was downloaded from the Amazon Web Services—AWS Terrain Tiles with a ground resolution zoom of 5, using the package *elevatr* in R [39]. To assess if birds fly at day or night during migration, we calculated a ratio of day over night time flight hours, corrected by available light and dark hours for each relocation, which was categorized as “day” or “night” according to geographic coordinates and date using the package *maptools* in R [40]. In this way, a flight ratio greater than 1 would indicate a greater use of daylight over nighttime hours whereas values less than 1 suggest flights are mostly nocturnal. Finally, tortuosity was quantified as a straightness index, which is the actual path followed by an animal divided by the length between starting and endpoints of a traveling segment [41, 42], by using the package *amt* in R [38].

Travel speed, average daily hours of flight, average day to night flight ratio, median flight height above the ground and route tortuosity measured as straightness were then included as response variables in linear mixed effects models. Although some response variables are correlated (see Additional file 2: Table S1), we decided to use separate models in order to assess the effects of predictors on each traveling behavior independently. As daily hours of flight and day to night flight ratios were calculated for each day, average values per traveling segment were used in the models (since a traveling segment can be longer than a day). In terms of flight heights above the ground, we used median values instead of averages to avoid using altitude means biased by extreme measurements of the GPS device (such as flight altitudes below the surface), but did not remove negative altitudes to avoid eliminating low altitude flights [43]. We decided to use average or median values during traveling segments for the latter three response variables in order to characterize behavior while birds are covering distance during migration (not only flight), avoid temporal and spatial autocorrelation in our models and use a spatial scale closer to that of the available environmental data. To assess individual variation in the five traveling behaviors, for each bird we calculated the percentage change from their overall average during the corresponding migratory season. Note that this approach aimed to characterize variation within individuals and those traveling behaviors that cannot have values lower than zero (median flight heights and average daily flight hours) have a lower bound in percentage changes. Also, we visually assigned broad geographic regions (northern seas in Europe, Europe, Sahara and Mediterranean crossings, African coasts and Sahel) to each traveling segment to compare observed traveling behavior values between regions (Additional file 1: Fig. S3).

### Annotation of tracks with model predictors

The five traveling behaviors were included in linear mixed effects models as response variables to tests the effects of weather conditions, foraging habitat availability, fuelling at stopover and the progress of the migratory journey. In terms of weather, segments were annotated with data specified by the altitude of each relocation point approximated to the nearest corresponding pressure level available (1000, 925, 850, 700, 600, 500, 400, 300 millibar) in the NCEP Reanalysis I dataset provided by NOAA/OAR/ESRL PSD, Boulder, Colorado, USA (http://www.esrl.noaa.gov/psd/). The variables included according to pressure levels were air temperature, wind direction and speed and vertical air flow. Additionally, total atmospheric precipitation, cloud cover and surface air temperature were also included as weather variables. Because the NCEP Reanalysis I dataset is available in daily 6-h time intervals (0:00, 6:00, 12:00, 18:00) and in a 2.5° × 2.5° grid, only the relocation points of the birds corresponding to those hours were used and the data were interpolated spatially to match the latitude and longitude of each position. Wind components (u and v) were assigned to points in which the bird was flying (as defined above) and so time was also interpolated for the annotation of tracks with wind data. Experienced tailwind and crosswind were calculated using the flight assistance as tailwind equation [44]:

$$fa_{tailwind} = y\,cos\theta$$

where θ is the angular difference between the direction of the bird’s movement and the wind and $y$ is the speed of the wind. Weather data and tailwind assistance were extracted with functions of the *RNCEP* package in R [45].

Foraging habitat availability during each traveling segment was quantified as the proportion of overlap with water bodies. We downloaded Terra and Aqua combined Moderate Resolution Imaging Spectroradiometer (MODIS) Land Cover Type (MCD12Q1) Version 6 data product with 500 × 500 m resolution [46] through the *MODIStools* and *MODIStsp* packages in R [47, 48] for 2018, which was the year with most tagged birds. The Annual International Geosphere-Biosphere Programme (IGBP) classification included in the MODIS product was used to distinguish water bodies by grouping the categories defined as water bodies (value 17, at least 60% is covered by permanent water bodies) and permanent wetlands (value 11, inundated lands with 30–60% water cover and > 10% vegetation) into a single variable of proportion of overlap with aquatic habitats. Proportion of overlap was calculated by counting the number of pixels within a buffer of 5 km on each side of a traveling segment that matched the grouped water categories and then dividing by the total number of pixels along the segment.

To consider the effects of fuel deposition rates, days spent at stopover before each segment were included in the model as an approximation to available fuel for migration. In autumn, Caspian terns leave breeding colonies and remain in nearby staging areas in the Baltic Sea. For this season we considered such periods to represent initial fuelling before first, longer distance migratory flights [as in 34]. However, fuelling before migratory flight in spring occurs within wintering areas and so was not directly available from tracking data. For this reason days spent at stopover was not included in models for spring. Finally, the days since the start of migration were also added as an explanatory variable, as a measure of the time elapsed during that migratory journey. This variable is associated with a progress in migratory season and could possibly reflect learning processes that may arise from experience as both juvenile and adult birds advance and improve their migratory performance [cf. 18].

### Linear mixed effects models

The five traveling behaviors characterized for each segment were then related to the aforementioned predictors as explanatory variables, and we included individual, breeding colony and year as random effects and age as a fixed effect in the full model (Table 1). Multicolinearity of explanatory variables was tested by estimating the variance inflation factor (VIF) and tolerance of each variable, and variables would be discarded if tolerance was under 0.20 (variable inflation factor > 5) using the *car* package in R [49]. Explanatory variables were scaled with one standard deviation and centered around their mean [50] and power transformations were used to obtain distributions closer to normality for response variables.

**Table 1:** Summary of response variables, predictors (fixed effects) and random effects used in linear mixed effects models of traveling behaviors

Types of variables	Variable names
Response variables	Travel speed (km/day), median flight height (m above ground level), average daily flight hours, average day to night flight ratio, straightness
Predictors	Proportion of overlap with water bodies, tailwind, crosswind, vertical flow, air temperature, surface temperature**, total precipitation*, cloud cover, days of migration, days at previous stopover, age (adult or juvenile)
Random effects	Individual

Predictors with one asterisk (*) were not included in spring models and those with two asterisks (**) were not included in both autumn and spring models to avoid multicolinearity of explanatory variables. Random effects of breeding colony and year are not shown in the table because these were removed for the second step of analyses according to model support and singularity outlined in the methods

The full models were tested with individual and all possible combinations of additional random effects (breeding colony and year). For each model, the Akaike Information Criterion corrected for small sample size (AICc) values were calculated and only the random effects of the model with lowest AICc value that did not lead to model singularity were selected for the second step of analysis. In the second step, the AICc and Bayesian Information Criterion (BIC) values and weights were calculated for all possible combinations of fixed factors to select the best model. Since no candidate model had a weight greater than 70%, parameters were estimated as the weighted averages of the set of models that represented 95% confidence weight [50] using the package *MuMIn* in R [51] by averaging by the zero method [52]. To distinguish effects of predictors on response variables that differed from zero, 95% confidence intervals (CI) for each predictor were calculated.

### Migratory activity according to time of day

To test if flight speed and height varied according to the time of day, we used linear mixed effects models with the nested random effect of traveling segment within individual and age as a fixed effect. Flight speed was calculated as the distance travelled divided by elapsed time and flight height was calculated from the recorded flight altitude minus the ground elevation for flight intervals as previously described. The time of day was divided into “night” and “day” according to the time of sunrise and sunset for each relocation using the package *maptools* in R [40], and “dawn or dusk” for relocations that were within the same hour of sunrise or sunset. We used likelihood ratio tests to determine if models were different from the null hypothesis using the *lmtest* package in R [53].

### Measure of habitat selection

As a measure of habitat selection during traveling segments, the overlap with water bodies for observed relocations was compared to that of simulated random points. First, for each relocation 99 random points were generated with the *amt* package in R [38] within a circular buffer that we created for three separate threshold radii: 35, 50 and 80 km. There are no studies on the perceptual visual range of Caspian terns, so we used values for the radius of circular buffers that would be within a theoretical range of vision defined by a simple geometric relationship between the height of an observer and Earth's surface as in [69]. For a bird flying at an altitude of 500 m, the range of vision would be up to 80 km [69]. Rather than only relying on the maximum distance of 80 km, we also tested circular buffers of 35 and 50 km radii, which may be more realistic distances if there are physiological constraints on vision or objects such as properties of the landscape that obstruct the visual target [70]. Second, within a 5 km radius of all observed and random points, the proportion of overlap with water bodies was measured. Third, the p-value for each observed relocation was calculated with a one-tailed test from the generated random distribution. With this approach, p-values lower than 0.05 were considered to indicate evidence for habitat selection at observed points because they had greater proportion of water bodies than expected by chance from the landscape.

To test if birds were in relocations with greater proportion of water bodies during the night, dusk/dawn or day and during flight or periods of stationary activity (i.e. not flying) in traveling segments, generalized linear mixed effect models with binomial distributions were fitted with the proportion of significant observations as response variable for each buffer (35, 50 and 80 km radii). The included random effect was individual, and fixed effects were time of day, flight or stationary activity and the interaction between the two. Flights were identified as previously defined (ground speed > 10 km/h at the beginning and end of 30 min intervals) and periods of stationary activity within traveling segments are those when the bird has slower ground speed (≤ 10 km/h). Assumptions of normality, equal variance and autocorrelation for gaussian models and overdispersion for binomial models were checked using the *DHARMa* package in R [54] and fitted using *lme4* in R [55]. All graphs and maps were developed in R [56] and maps used *rnaturalearth* base layers [57].

## Results

Complete migratory tracks were recorded for 39 Caspian terns (12 adults, 27 juveniles) in autumn and for 12 adult Caspian terns in spring (217 and 42 traveling segments for autumn and spring, respectively).

### Variation in traveling behavior

**Fig. 1:**
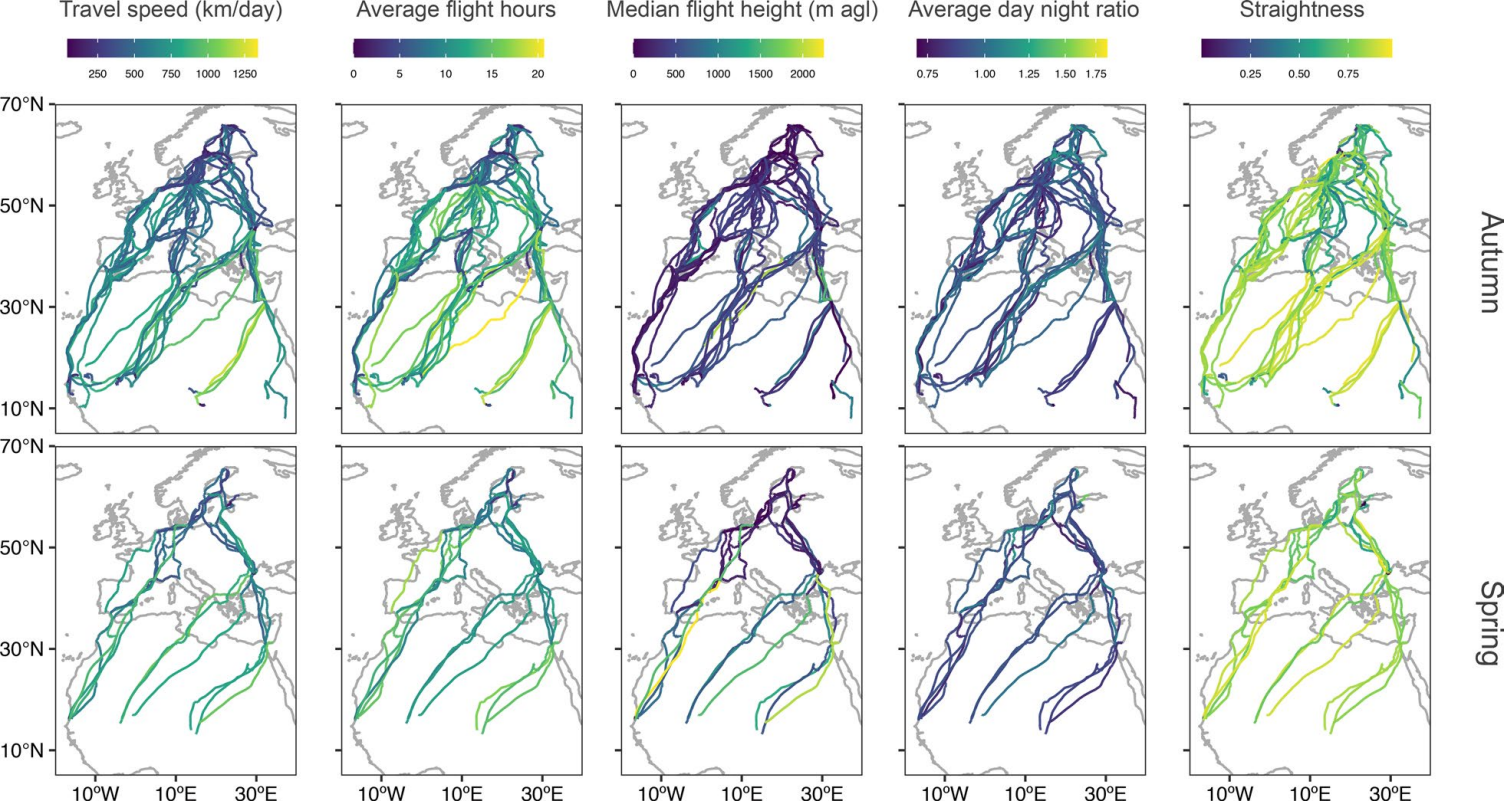
Values of five measured traveling behaviors along the route of autumn (top row, 12 adults and 27 juveniles) and spring (bottom row, 12 adults) migration of tracked Caspian terns breeding in the Baltic Sea. Note that the color scale for each traveling behavior varies according to scale of observed values, and in the  panel of average day to night flight ratio the color scale is logarithmic to aid visualization. See Additional file 1: Fig. S6 to consult percentage individual variation in traveling behaviors

Variation in the five measured traveling behaviors was on average between 10 and 155% changes from overall values calculated for each individual, with median flight heights having the greatest percentage variation and average day to night flight ratios the lowest variation in comparison to other variables of traveling behavior for both seasons (Fig. 1, Additional file 2: Table S2). All traveling behaviors varied according to broad geographic regions (likelihood-ratio tests p < 0.05, Additional file 2: Table S3). The general pattern we found was that in northern Europe Caspian terns lowered travel speeds (290 (136 sd) and 360 (89 sd) km/day in autumn and spring, respectively), median flight heights (127 (107 sd) and 91 (58 sd) m above ground level), average daily flight hours (6 (3 sd) and 7 (2) h) and straightness (0.63 (0.17 sd) and 0.67 (0.14 sd). Also, they increased average day to night flight ratios (1.01 (0.22 sd) in both seasons) in this region. In contrast, birds had higher travel speeds (849 (231 sd) and 844 (114 sd) km/day in autumn and spring, respectively), median flight heights (539 (400 sd) and 1267 (570 sd) m agl) and average daily flight hours (15 (3 sd) and 13 (2 sd) h) over the Mediterranean Sea and the Sahara Desert for both seasons. Notably, the birds that followed the coasts in West Africa or the Red Sea in the east in their southwards journey during autumn also had increased travel speeds (726 (95 sd) km/day) and flight hours (11 (3 sd) h), but lower flight heights (184 (127 sd) m agl) in comparison with those that crossed the desert far away from the coast (Fig. 1 and Additional file 1: Fig. S6, Additional file 2: Table S4).

**Fig. 2:**
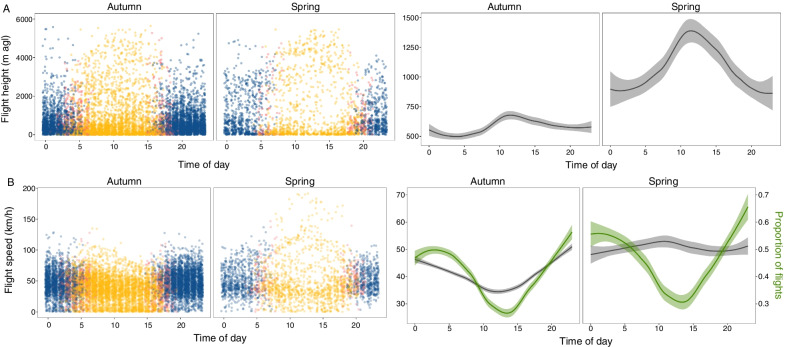
Flight height above ground (**A**–**B** and **E**–**F**) and flight speed (**C**–**D** and **G**–**H**) for flights during traveling segments for 39 tracked Caspian terns during autumn (**A**, **C**, **E**, **G**) and spring (**B**, **D**, **F**, **H**) migration. Flights were defined when birds had an instantaneous speed greater than 10 km/h both at the start and end of 30-min intervals. Panels on the top row of the figure show all recorded flights, with nighttime (blue), daytime (yellow), and dusk or dawn (pink) indicated by colors (**A**–**D**). Extreme values of flight heights above the ground that resulted from unreliable GPS reads (> 6,000 m and with unrealistic climb rates or < 0 m agl) were excluded from the graph. The bottom row of the figure shows the smoothed lines of average speed and height above ground (**E**–**H**), with lines representing average values and shaded areas the 95% confidence interval. Note that since the graphs of the bottom row represent averages the scales of the y axis between top and bottom panels differ. The proportion of recorded flights was calculated as the number of identified flights compared to total relocations for each hour, and it is included as a green line in panels **G** and **H** and referenced by the right axis

Migratory flights occurred mostly during the night, but daytime flights were also present. Likelihood ratio tests showed that there were significant differences in flight speeds and heights between the day, dawn or dusk and nighttime. Compared to dawn or dusk and the night, during the day birds flew at greater heights in both seasons (χ^2^ = 22.57, d.f. = 7, p < 0.0001 and χ^2^ = 74.97, d.f. = 6, p < 0.0001 for autumn and spring, respectively) and at lower flight speeds in autumn (χ^2^ = 567.5, d.f. = 7, p < 0.0001). In contrast, in spring there were very high daytime flight speeds in the northbound Sahara crossing, and so flight speeds were on average higher during the day compared to dawn or dusk and night (χ^2^ = 56.999, d.f. = 6, p < 0.0001). There were also differences between the seasons: birds flew on average higher (χ^2^ = 335.39, d.f. = 4, p < 0.0001) and at greater flight speeds (χ^2^ = 238.37, d.f. = 4, p < 0.0001) during spring in comparison to autumn (Fig. 2).

### Predictors of traveling behavior

**Fig. 3:**
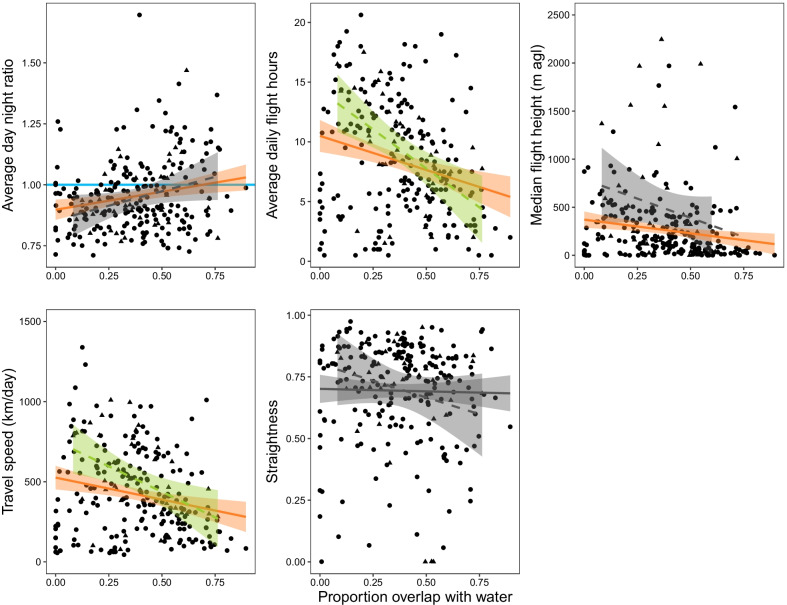
Regression between proportion of overlap with water bodies and five variables related to a fly-and-forage strategy calculated for traveling segments of autumn (solid line) and spring (dashed line) migration of tracked Caspian terns (12 adults and 27 juveniles represented as circles in autumn and 12 adults as triangles in the spring). Proportion of overlap with water bodies was measured as the proportion of pixels in a 5 km buffer around traveling segments with identified aquatic habitats according to MODIS land covers (see methods). Orange (autumn) and green (spring) lines signal the variables for which the proportion of overlap with water was a predictor with effects that did not overlap with zero in a 95% confidence interval (CI), while grey lines show predictors with effects that overlapped with zero (95% CI). Horizontal light blue line in the graph of day to night flight ratio indicates when the ratio value is equal to 1, since a value greater than 1 represents migratory flight predominantly during the day and less than 1 during the night. See Additional file 1: Fig. S7 for correlation graphs of all explanatory variables with effects distinguishable from zero

**Fig. 4:**
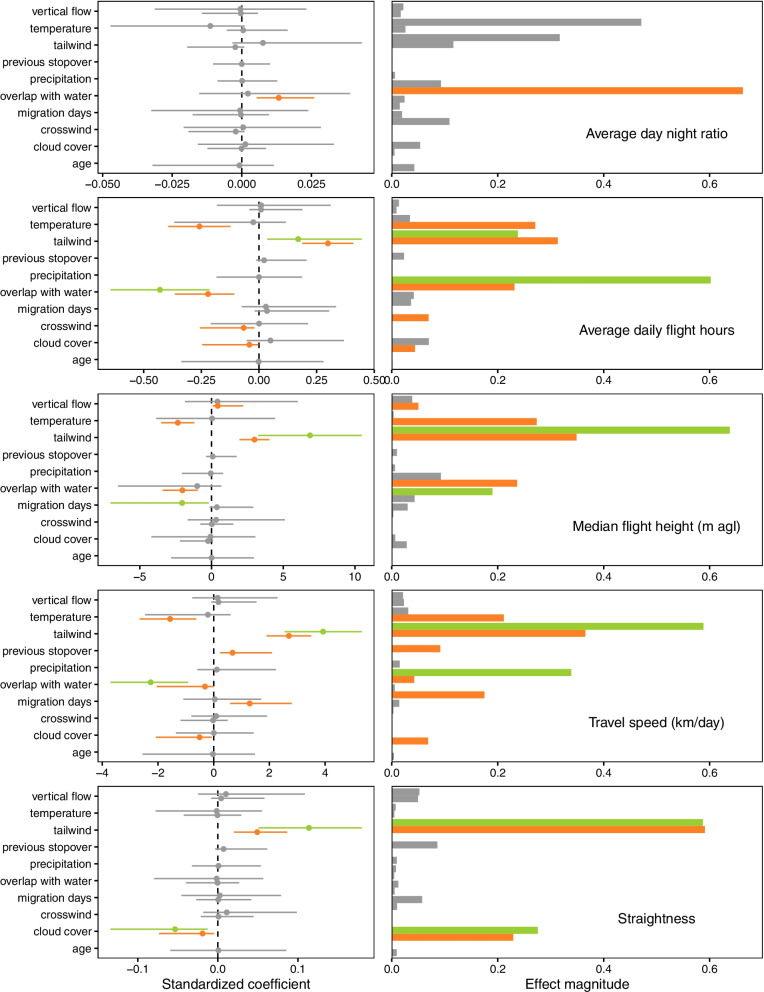
Standardized coefficient estimates and compared effect magnitudes of predictors averaged across linear mixed models for five traveling behaviors as response variables: average day to night ratio (**A**), average daily flight hours (**B**), median flight height above ground (**C**), travel speed (**D**) and straightness (**E**). Standardized estimates are shown as points, with horizontal lines indicating 95% confidence intervals (CI). Predictors with estimates different from zero (95% CI) are shown in colors, green for spring migration and orange for autumn. Effect magnitudes were compared by dividing the estimated coefficient of each predictor by the sum of all coefficients. Note that age, time spent at previous stopover and precipitation were not included for spring migration, since only adults migrated back to the breeding grounds in this season, the first stopovers at the wintering areas were not possible to estimate from the data and precipitation was removed from spring models to avoid correlation between explanatory variables. Variables are organized in descending alphabetical order

Available foraging habitat, measured as the proportion of overlap with water bodies, was a predictor with negative effects on travel speed, flight height and daily flight hours, and positive effects on day to night flight ratio in autumn (effects on four out of five traveling behavior variables different from zero in a 95% CI). In spring, proportion of water bodies was also negatively related to travel speed and daily flight hours (effects on two out of five variables different from zero in a 95% CI). Remarkably, the proportion of overlap of routes with water bodies was the only variable with a distinguishable effect on the calculated day to night flight ratio in autumn, yet none of our predictors had strong effects on day to night flight ratios in spring (Figs. 3 and 4, Additional file 2: Table S5).

In the case of the response variable related to tortuosity measured as straightness, the effect of the proportion of overlap with water was indistinguishable from zero. Instead, tailwinds and cloud cover were the only predictors with strong effects on straightness (Fig. 4). Tailwind was the only other explanatory variable besides habitat availability with distinguishable effects on four traveling behaviors, and in all cases but one (daily flight hours in spring) it was the strongest predictor.

In all models, the effects of precipitation and age were indistinguishable from zero. The explanatory variables with high VIF due to correlation were surface temperature and air temperature in autumn and surface temperature, air temperature and precipitation in spring (Additional file 2: Table S8, Additional file 1: Figs. S4 and S5). We decided to remove surface temperature as an explanatory variable in models for autumn and surface temperature and precipitation for spring models. Air temperature was preferred over surface temperature because it may be more directly related to the conditions the bird is experiencing according to altitude. For all models, individual was included as the single random effect since it resulted in models with lower AICc values compared to all possible combinations with additional random variables. In three models for traveling behaviors for spring other combinations of random effects (individual + breeding colony or individual + year) had a lower AICc value but resulted in a singular fit, so individual was included as the single random effect (Additional file 2: Table S7).

We were not able to calculate days at previous stopover for spring migration because the first stopovers occurred inside the wintering areas and were not detected from our data. Also, age was not a factor included in the models in spring since only adults performed complete migrations back to the breeding grounds. For the presentation of our results and discussion, we refer to results from estimates calculated using the BIC criterion for model selection because in all cases they resulted in less models needed for 95% weights. Model estimates calculated by averaging according to AICc show similar general patterns, and can be consulted in Additional file 2: Table S6.

### Measure of habitat selection

**Fig. 5:**
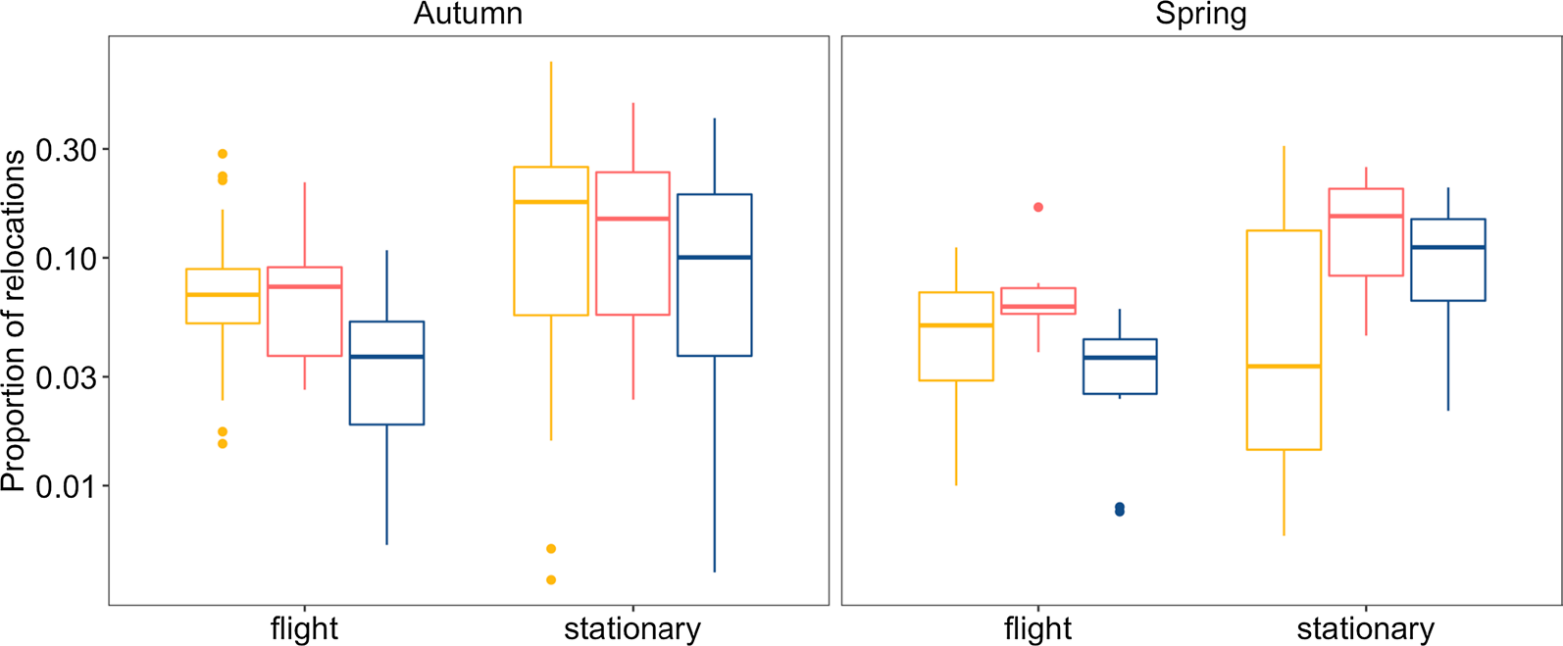
Proportion of relocations with significantly more available foraging habitat measured as proportion of overlap with water bodies, according to periods of migratory flight (ground speed > 10 km/h at both the start and end of 30-min intervals) and stationary relocations (ground speed < 10 km/h) within traveling segments for tracked Caspian terns (27 juveniles in autumn and 12 adults in autumn and spring). Color of boxplots indicates time of day, with yellow for daytime, pink for dawn/dusk and blue for nighttime. The middle thick line of the boxplots is the median, hinges of the box are the first and third quartiles, lines extend 1.5 of the inter-quartile range and outliers are represented by points. Note that the scale of the y-axis is logarithmic to aid visualization. See Additional file 1: Fig. S9 for boxplots of 35 and 80 km buffer distances

All individuals in both seasons had migratory tracks with relocations that had a greater proportion of water bodies in their surrounding area than expected by chance from the landscape, for all three buffer thresholds of 35, 50 and 80 km radii (Additional file 1: Fig. S8). Excluding those relocations that were over areas with a homogeneous landscape (e.g. the sea or desert, with the presence of water bodies being complete or non-existent, respectively), between 35 and 49% of migratory traveling days had at least one relocation with significantly more available habitat than its surroundings in spring and autumn, respectively. In days when there was evidence of habitat selection, birds spent an average of 2.8 to 5.7 h per day in such areas in spring and autumn, respectively. For all three buffer threshold distances, time of day and flight or stationary activity were significant explanatory variables in both seasons (Additional file 2: Table S9). Caspian terns were more frequently in areas with greater habitat availability than that offered by chance from the landscape during day and dawn or dusk in comparison to nighttime and in periods of stationary periods instead of flight, yet the proportion of relocations with greater proportion of water bodies than expected by chance in the landscape increased during nighttime when birds were in stationary activity periods (i.e. not flying during traveling segments) (Fig. 5 and Additional file 2: Table S9). Additionally, the number of relocations that evidenced habitat selection and the time spent at these points decreased as the manipulated buffer distance increased (Additional file 1: Fig. S10).

## Discussion

Our results show within-season variation in the migratory traveling behaviors of a bird that depends on water bodies for food yet manages to cross landscapes that have marked differences in available foraging habitat. Migratory behaviors of Caspian terns are predominantly associated with experienced wind conditions, as has been reported for several other species [5, 7, 11], but the observed variation in behaviors was also related to the proportion of overlap of tracks with bodies of water. When the proportion of overlap with possibly suitable habitat was greater, in both seasons Caspian terns lowered travel speed and spent fewer hours of the day advancing in migratory flight. In autumn, terns also flew at lower heights above the ground and dedicated more daylight hours for flight in comparison to nighttime hours as the proportion of water increased. Moreover, birds reduced flight speeds during daytime, but contrary to our expectations increased flight heights during the day. We also found evidence for habitat selection along the migratory route within traveling segments for every tracked individual, including adults and juveniles. Caspian terns were in areas with greater proportion of available habitat compared to that expected by chance from the landscape, and more frequently so when there was daylight and in periods of stationary activity of traveling days in comparison to flights. Variation in traveling behaviors suggests how foraging is limited by the availability of aquatic habitats, as had been previously shown for ospreys [26]. Also, behavioral adjustments coincide with what would be expected from a fly-and-forage migrant that is fuelling during traveling days, since foraging requires decreased travel speeds, interruptions to migratory flight and flights closer to the ground to search for food as well as light for the visual detection of prey [26, 27, 29].

Alerstam (2009) had previously hypothesized that a fly-and-forage strategy could benefit from diurnal migratory activity because light is needed to visually identify foraging habitat and prey. In the case of Caspian terns, we found that they mostly fly at night during migration but are able to alter daily migratory schedules. Ospreys have also been shown to alter daily routines during migration, changing the frequency of migratory flights according to time of day [26]. The difference with Caspian terns is, however, that ospreys do not use nighttime for migratory flight because they are soaring raptors [26]. We found that during autumn increasing diurnal over nocturnal migratory flight activity of Caspian terns was associated with more available foraging habitat along the route and the proportion of overlap with water bodies was the only predictor with an effect distinguishable from zero (95% CI) on day to night flight ratios in our models.

The ability to adjust daily migratory schedules has been previously demonstrated for songbirds that extended their nocturnal flight into daylight hours to cross the Sahara [58]. Larger bird species have also been reported to cross ecological barriers during the day [24], as we also found from the daytime crossings over the desert and sea for Caspian terns. Despite this diurnal activity over areas with little to no foraging habitat, the general trend we found was the opposite: increased diurnality compared to nocturnal flight in this species has a positive correlation with habitat availability during migration. When the overlap with water was greater than 61% in spring and 69% in autumn, day to night flight ratios surpassed the value of 1, indicating more day over nighttime flight. The alternative explanation to this pattern could be that during daytime flights birds select suitable habitats, which would also suggest they have foraging opportunities while advancing on migration and change their behavior with the presence of available habitat. Circadian rhythms governing migratory schedules have been proposed to be under endogenous regulation and have a strong genetic basis [59, 60] yet internal clocks may need to be in constant shift for navigation purposes (see [2] for a review). Here we give evidence for variation in daily migratory rhythms, but future research is still needed to understand causal relationships and the proximate mechanisms to explain how this may be achieved.

We also found that terns reduced flight speeds during daytime flights in comparison to dusk or dawn and night, as has been detected in other fly-and-forage migrants [61]. The exception was when adults also had very high diurnal flight speeds in spring in the northbound crossing of the Sahara with favorable tailwinds at an average of 3800 m above the ground. Notably, when crossing the Sahara Desert and the Mediterranean Sea the birds adopted a “sprinting” migration with increased travel speeds, flight heights and daily flight hours, resulting in a mixed migratory behavior that varies with available habitat [26] and combines foraging along the route with stopovers in freshwater and coastal ecosystems [34]. Mixed strategies that include both stopovers and fly-and-forage have been previously recognized for a handful of species including ospreys [26, 27], Eleonora’s falcons [61], lesser black-backed gulls [30], Cory’s shearwaters [62], common swifts [63, 64] and bank swallows [65]. We foresee that more fly-and-forage migrants will be described as tracking technologies advance and continue to give new insights into the behavior of migratory birds.

Contrary to what would be expected from a fly-and-forage migrant, flight heights during the day were actually greater than those during dawn or dusk and night. Daytime migratory flights may also favor the use of thermals to climb altitudes by soaring [29]. Although Caspian terns are not known to soar in their breeding grounds, their wing aspect ratio (12.6 [36]) could allow for soaring and represent an effective way to increase flight altitude [66]. However, the occurrence of warm air columns was not related to daily migratory schedules and was related to flight height in autumn but not in spring.

For autumn, other variables related to flight height besides vertical air flows were temperature, proportion of overlap with water bodies and tailwind. At higher altitudes, birds may avoid very high surface temperatures and evaporative loss [11], find favorable winds for flying [5, 11] and possibly decrease predation risk and augment their range of vision [67]. The only predictor with strong effects on flight height common to both seasons was tailwind, which suggests that finding favorable air flows might be the main factor behind the selection of flight altitudes, particularly in spring, when flight altitudes and speeds were higher than in autumn. Previous research has demonstrated that the selection of flight altitudes may be key in achieving optimal wind support and play an important role in successfully surpassing ecological barriers [22]. Also, a recent study on European nightjars has shown that altitude shifts are frequent during migration [68], supporting the high within-season variation in flight heights we found for Caspian terns.

The only traveling behavior variable we measured that was not related to foraging habitat availability in any season was route tortuosity. Detours may be avoided by Caspian terns while foraging on the move to reduce related costs. According to [27], fly-and-forage is advantageous if the benefits (*b*) are greater than the product of the costs (*c*) and power ratio (*p*), which is the power required for flight over the rate of fuel deposition [3], as follows:

$$b > c*\left( {1 - p} \right)/p$$

Since this species has a specialized diet [35], time must be dedicated to finding suitable habitat, locating a fish in the water, diving to catch it, then handling and digesting their prey. To not lower traveling speed even further, Caspian terns may fly-and-forage using habitat that is already part of the route between stopovers but not go off course to search for foraging areas. Route tortuosity was less flexible than most other traveling behavior variables and we found that the number of relocations and the time spent in habitats with significantly more water compared to random relocations decreased as we increased the buffer distance for the random points. We recognize that the thresholds of 35, 50 and 80 km radii buffers were arbitrary and to determine more appropriate distances the perceptual range of vision for this species should be measured and the properties of the landscape that may obstruct visual detection should be considered [70]. However, our experimental variation of the buffer distances and obtained results support the theory that the cost of visiting a foraging area farther off may outweigh the benefit of fuelling, and presumably not be an optimal decision for long-distance migrants that need to keep migratory schedules within their annual cycles [27]. The combination of time at stopovers with exploration of foraging areas in a shorter distance migrant, the subspecies of lesser black-backed gull *(Larus fuscus*) breeding in the Netherlands, has been shown to result in one of the lowest overall migratory speeds recorded for any species (44 and 98 km/day in autumn and spring, respectively) [30]. Although fly-and-forage may optimize time by reducing the need for long stopovers [28], it can also be optimal for energy rather than time if extensive foraging occurs along the route or stopovers are also included [30], or a combination of both. In our study species, evidence of behavior related to fly-and-foraging was present in both seasons. However, birds substantially reduce the number of stopovers and time spent at stopovers in spring compared to autumn [34], suggesting that foraging along the route possibly optimizes energy in autumn and time in spring.

Increased detours during migration were instead related to tailwind and cloud cover in both seasons, with straighter routes occurring with clearer skies and increased tailwinds. The relationship between cloud cover and route tortuosity for complete migratory tracks has not been reported before for any other bird species, yet our findings support previous work with songbirds that has found greater departure probabilities [8, 71] and more oriented departures [71, 72] in clear skies. Such studies suggest the relevance of visibility for detecting celestial cues, such as the starry sky and the position of the sun for orientation [73–75]. Experimental approaches are needed to distinguish if straightness decreased with greater cloud cover because it affected orientation or if it is also related to weather conditions (not captured by the other climatic variables included in the models such as air temperature and precipitation) that caused birds to make detours related to avoiding inclement weather [5, 76]. Also, cloud cover could be related to the visual detectability of fish through the amount of light that is reflected by the water surface or the prey itself, but this remains largely understudied.

Good visibility is also important to detect landmarks and other beacons for navigation [77] as well as control flight speed, direction, and altitude through optical flow [78, 79] and correct for drift [80]. Water bodies may be useful beacons, and previously it had been suggested that Caspian terns follow rivers across Europe and coasts along their route to migrate [32]. Our finding that birds are selecting aquatic habitats 35 to 49% of their total traveling days supports this idea. However, this number may underestimate habitat selection because our methods did not adequately capture coasts as relocations that had more suitable habitat than the surroundings (see Additional file 1: Fig. S8), even though shallow waters are ideal foraging areas and Caspian terns have a tendency to follow coasts during migration. Topography could also play a role in site selection and be confounded with the presence of water in low elevations such as river valleys, yet the patterns found between proportion of overlap of water bodies with traveling behaviors indicate that foraging most probably takes place near suitable habitat. However, we highlight that our method to detect habitat selection was a useful but coarse approximation, since suitable foraging habitat for this species may also be determined by finer-scale characteristics such as water depth, water quality, vegetation covers [81], and fish species richness and abundance [82], as well as be affected by predation risk.

Age was not an explanatory variable with strong effects in any of our models for autumn migration, although experience presumably plays an important role in migratory behavior [18]. Juvenile Caspian terns are known to migrate in the company of adults [32, 34, 83] so inexperience is probably counterbalanced by their guidance and learning processes along the route, which were not successfully captured by days of migration as a predictor included in our models. It will be interesting to analyze first spring northbound migrations of juveniles and study possible learning processes and age-related differences in the future. Another variable included in the models that needs further improvement is the available fuel birds have to power migration, since the days at previous stopover was not a predictor with strong effects for any traveling behavior in autumn and it was not possible to calculate for the first fuelling sites in spring. Available energy is key for understanding bird migration [3] and needs to be quantified in another way to have a more comprehensive picture in this regard.

## Conclusions

Our study provides new insights into how traveling behaviors are associated to available foraging habitat during bird migration, not only when birds face the challenges of ecological barriers but along the entire migratory route. Furthermore, we give evidence for foraging and habitat selection outside of stopover areas, thus illustrating a mixed migratory strategy for a bird that depends on aquatic ecosystems but manages to migrate over changing landscapes.

In this sense, Caspian terns challenge dichotomies between land and seabirds in the study of avian migration in two ways. First, although Caspian terns from the Baltic breed on rocky islands in the sea, these birds exploit freshwater ecosystems outside of their breeding range to fuel migration and spend wintering months [34], so they cannot be strictly considered as seabirds during their full annual cycle. Second, a mixed migratory strategy necessarily combines behaviors associated with both stopover use and fly-and-forage. This means that migratory behaviors usually thought to be unique to landbirds, such as foraging by day and flying at night, fuelling at stationary periods and crossing ecological barriers, are also present. Within-season variation in behaviors related to either alternating flight with stopovers and fly-and-forage strategies may affect the benefits and costs of migration in relation to the experienced environmental context [27] and therefore also question placing species in strict categories of migratory behaviors.

Finally, our findings also highlight the importance of protecting habitat in migratory flyways. The population of Caspian terns in the Baltic Sea has declined since the 1970s [84]. Possible threats have been identified in breeding [85, 86] and wintering areas [33, 87, 88], but we stress that maintaining healthy aquatic ecosystems, particularly in Europe, is also crucial for this species to complete migration because it forages along the route.

## Supplementary Information

**Additional file 1.** Supplementary figures.

**Additional file 2.** Supplementary tables.

## Data Availability

Data is stored in the Centre for Animal Movement Research at Lund University in Sweden (< www.canmove.lu.se >) and will be made available in the Movebank Data Repository following a 1-year embargo at https://doi.org/10.5441/001/1.hg1v55ct (Åkesson et al., 2022).
