# Publisher Correction to: Foraging on the wing for fish while migrating over changing landscapes: traveling behaviors vary with available aquatic habitat for Caspian terns

**DOI:** 10.1186/s40462-022-00321-w

**Published:** 2022-06-03

**Authors:** C. Rueda-Uribe, U. Lötberg, S. Åkesson

**Affiliations:** 1 Department of Biology, Centre for Animal Movement Research, Lund University, Ecology Building, 223 62 Lund, Sweden; 2 BirdLife Sweden, Stenhusa gård, Lilla Brunneby 106, 386 62 Mörbylånga, Sweden

## Correction to: Movement Ecology (2022) 10:9. 10.1186/s40462-022-00307-8

The original publication of this article [1] contained an incorrect version of Figs. 2 and 4 which did not contain the panel indications.

The original article has been updated with the corrected figures. The publisher apologizes for the inconvenience caused.
