# Supplementary material for: Foraging on the wing for fish while migrating over changing landscapes: traveling behaviors vary with available aquatic habitat for Caspian terns
Source: Mov Ecol. 2022 Mar 2;10:9. doi: 10.1186/s40462-022-00307-8 (PMC8892754; doi:10.1186/s40462-022-00307-8)
Supplement: Supplementary file 1 — Additional file 1. Supplementary figures. [file 40462_2022_307_MOESM1_ESM.docx]

Additional file 1: Figures


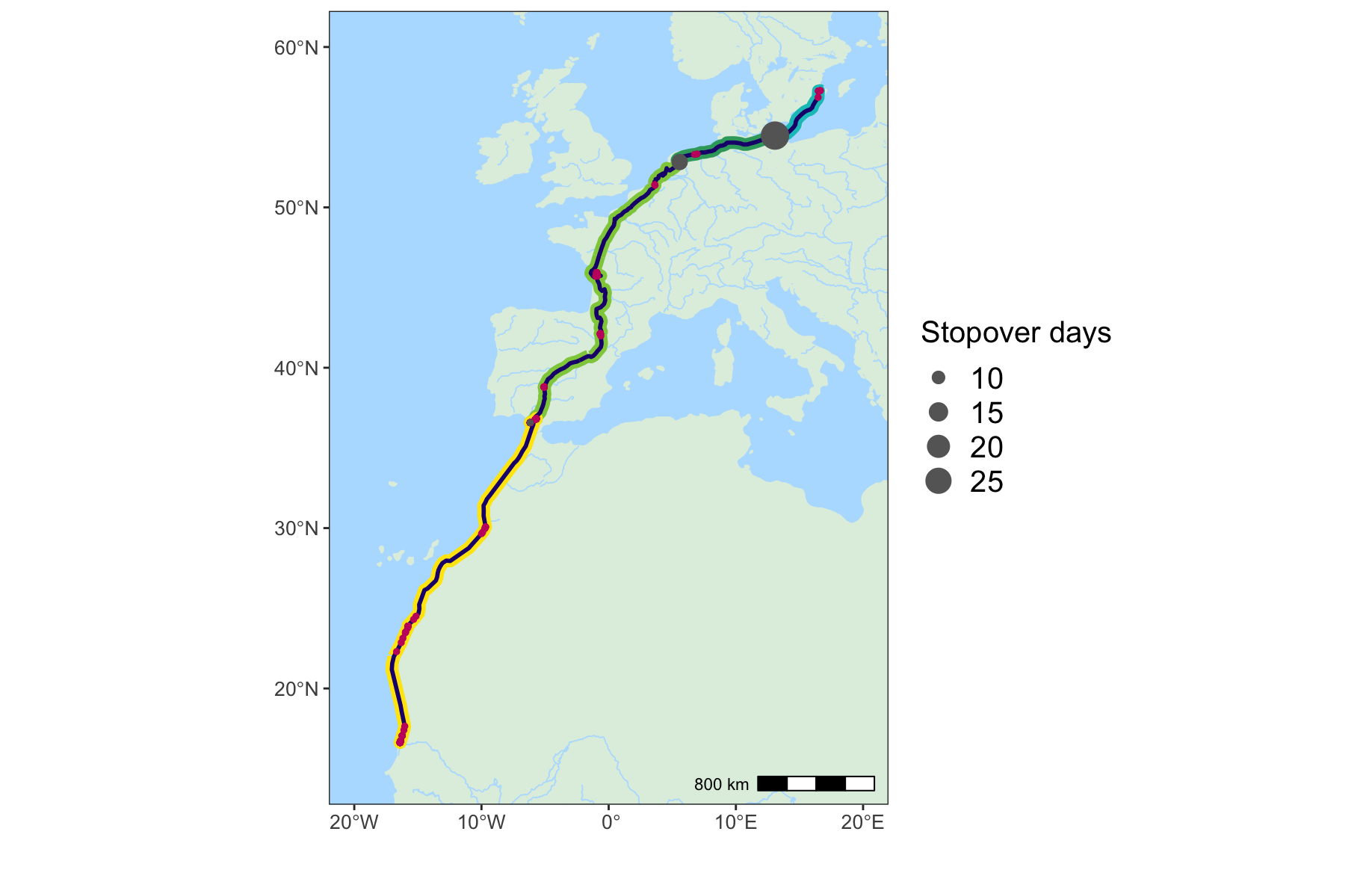


Figure S1. Example track of one individual Caspian tern during autumn migration illustrates how traveling segments correspond to periods where birds advance in migratory distance between stopovers, indicated with changing colors around the track (blue, dark green, light green and yellow for the four traveling segments of this example). Stationary relocations when birds are possibly not flying (instantaneous groundspeed <= 10 km/h) are indicated in red. Stopovers are signaled as grey circles and the size of circles shows the number of days spent at stopover. Base layers for landmasses in light green and river and lakes shown in blue were downloaded from *rnaturalearth*. To see segments of all tracked individuals, refer to Fig. S2.


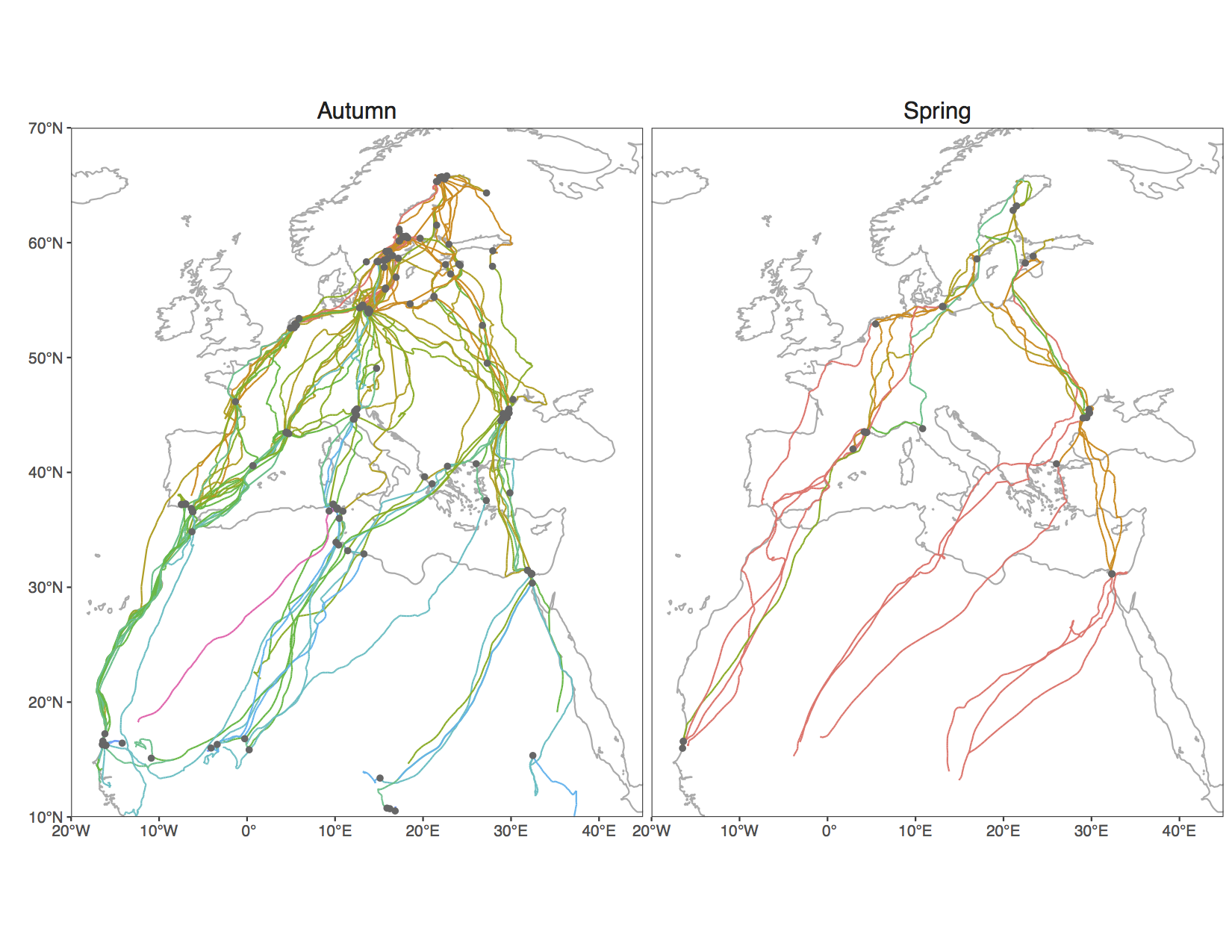


Figure S2. Traveling segments of all tracked Caspian terns during autumn (12 adults and 27 juveniles) and spring (12 adults) migration. Different segments are distinguished by different colors within individuals. Stopovers separating traveling segments are indicated with grey circles.


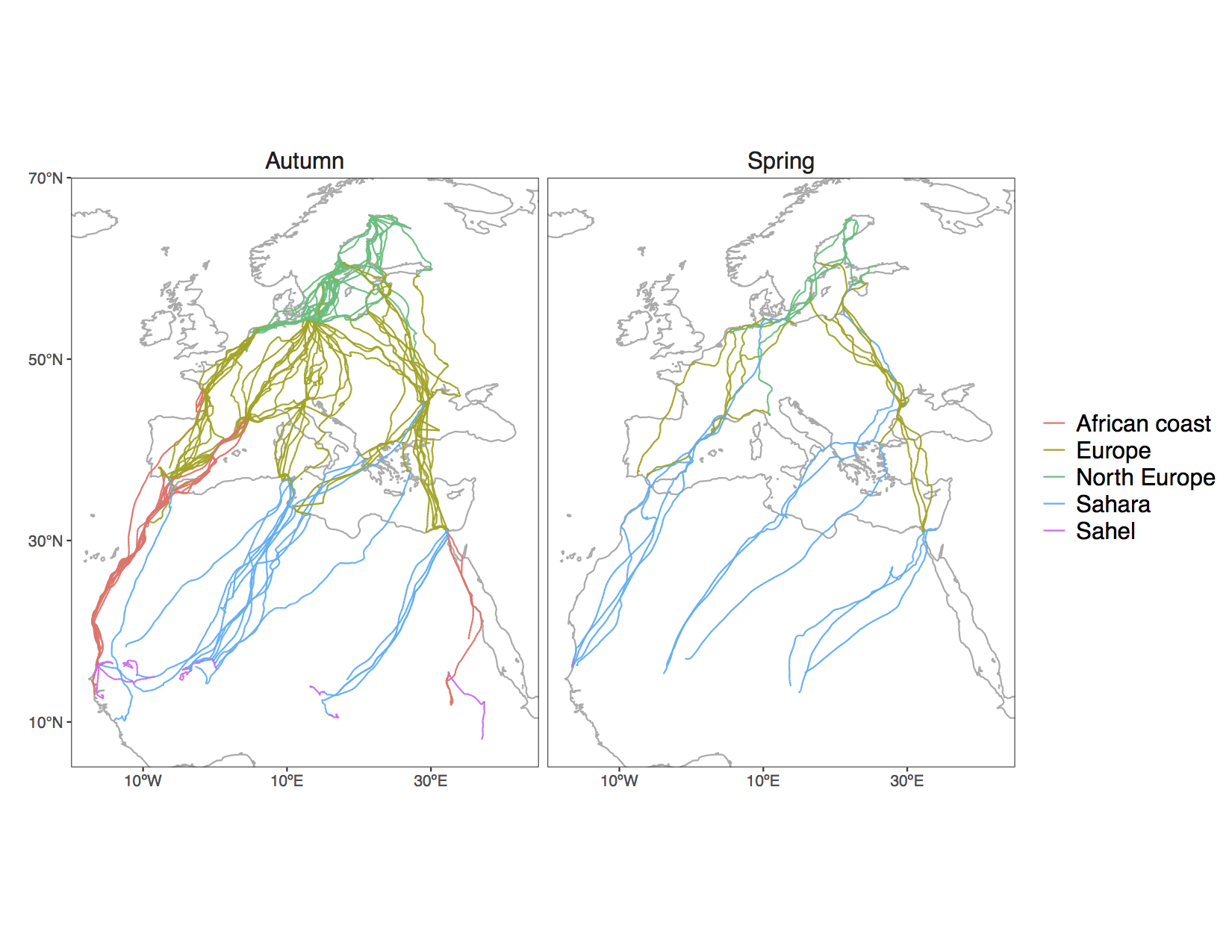


Figure S3. Broad geographic regions assigned to traveling segments during autumn (12 adults and 27 juveniles) and spring (12 adults) migration of tracked Caspian terns. Note that in spring some individuals did not stop over until reaching the Baltic Sea.


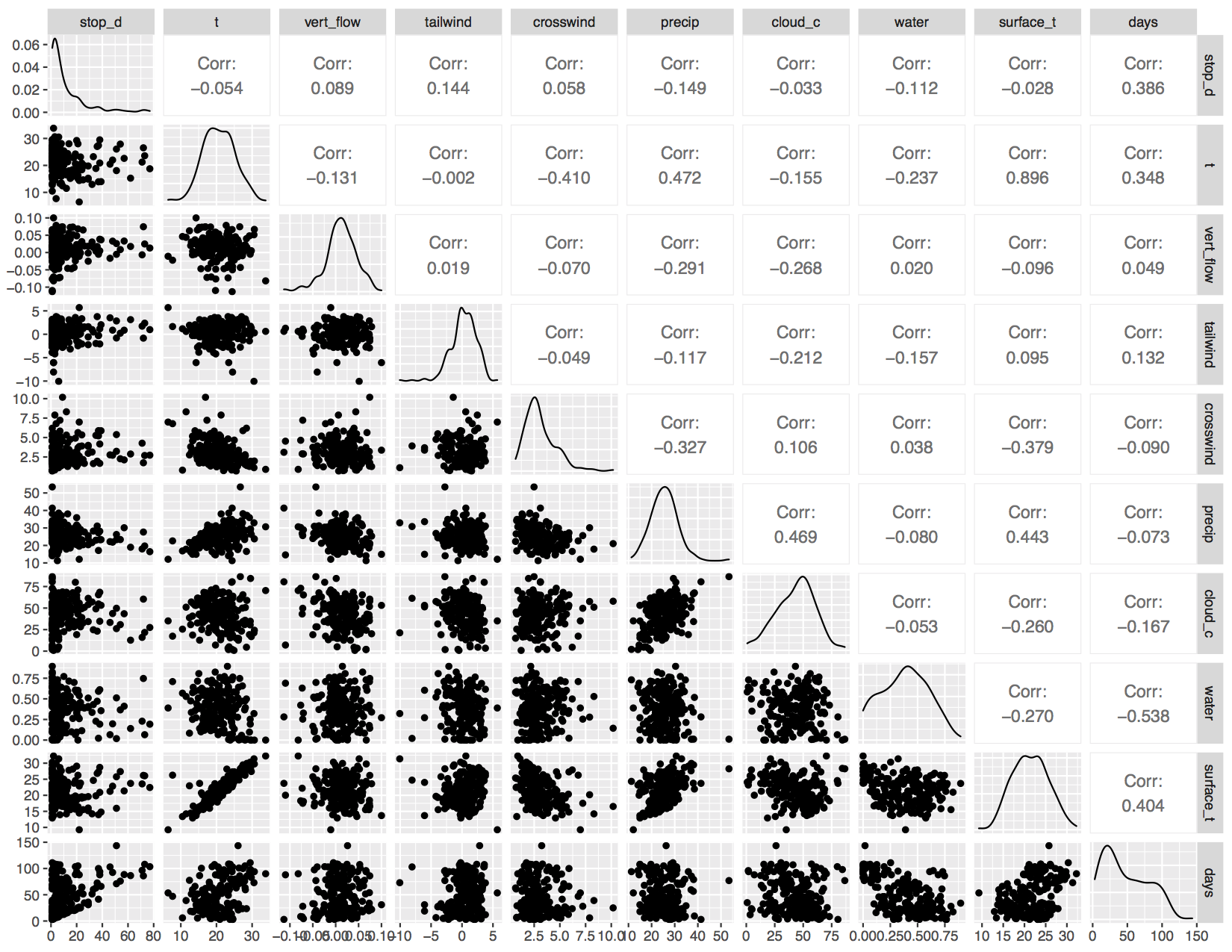


Figure S4. Multicolinearity assessment of explanatory variables included in the model for autumn migration testing association with five traveling migratory behavior variables related to a fly-and-forage strategy for tracked Caspian terns (12 adults and 27 juveniles in autumn).


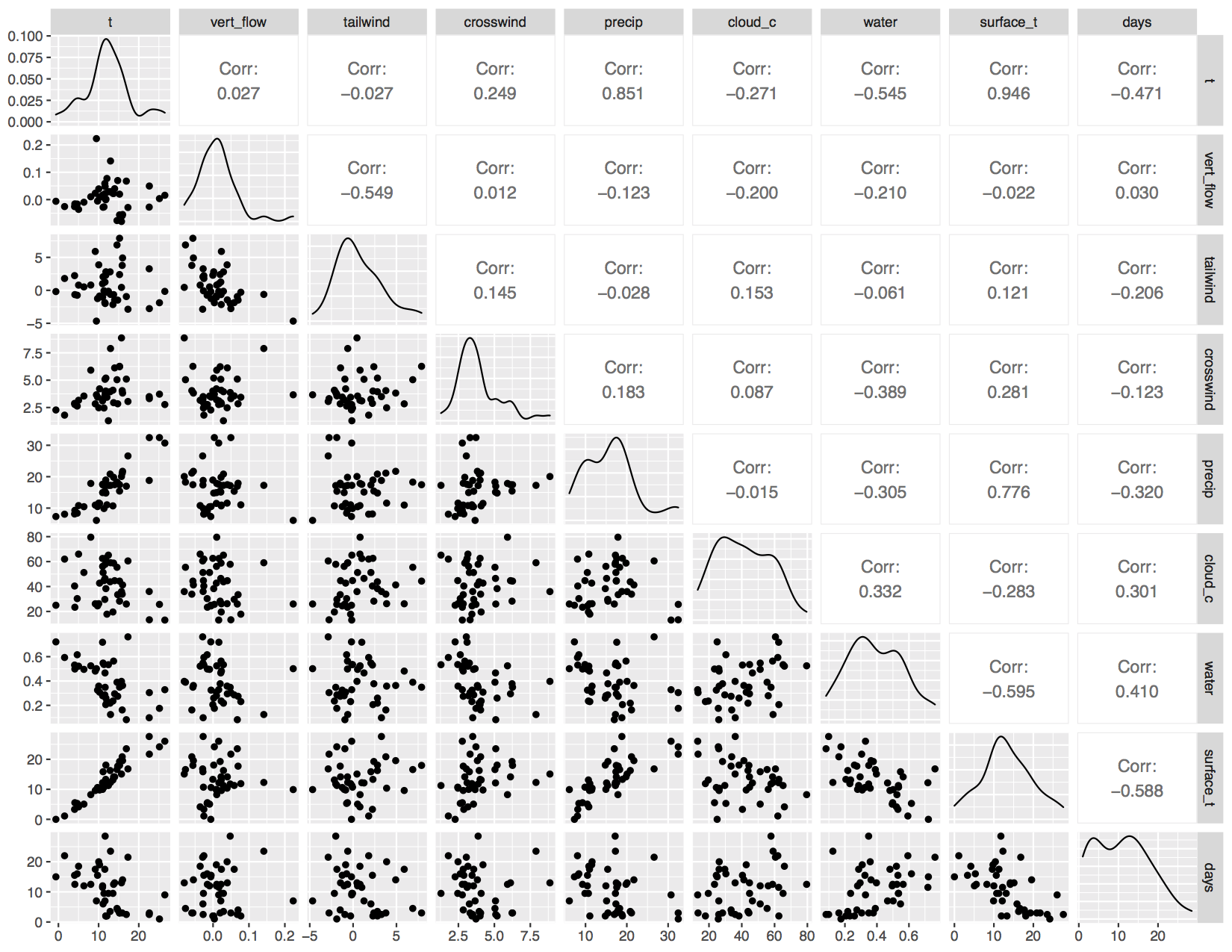


Figure S5. Multicolinearity assessment of explanatory variables included in the model for spring migration testing association with five traveling migratory behavior variables related to a fly-and-forage strategy for tracked Caspian terns (12 adults in spring).

Figure S6. Individual variation of traveling behaviors along the route of autumn (top row, 12 adults and 27 juveniles) and spring (bottom row, 12 adults) of tracked Caspian terns breeding in the Baltic Sea. Percent change is calculated as the difference of value for traveling segment from the overall average during the corresponding migratory season (autumn or spring) for each individual. Note that the color scale is logarithmic to aid visualization.


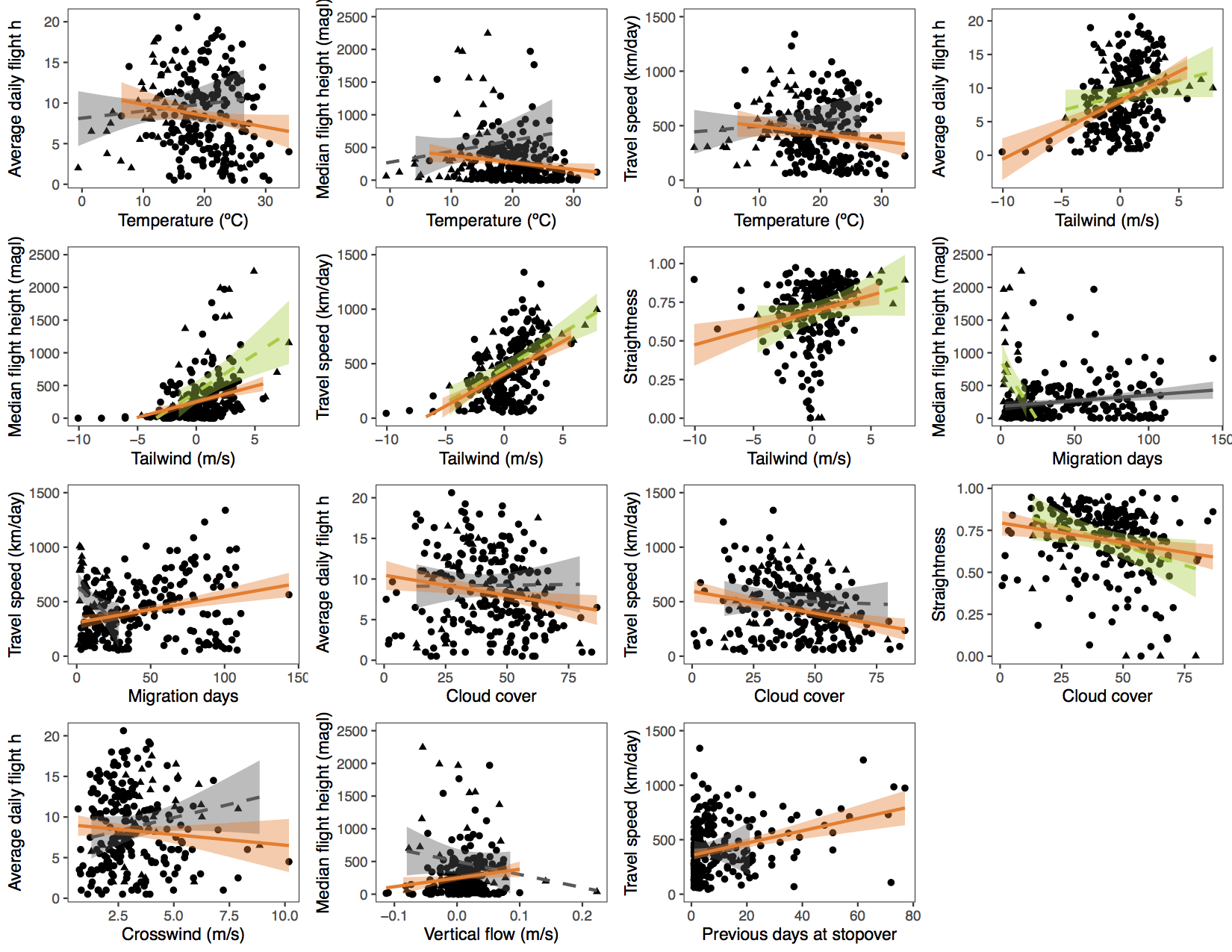


Figure S7. Regression of significant predictors for traveling migratory behavior variables related to a fly-and-forage strategy in autumn (solid line) or spring (dashed line) migration of tracked Caspian terns (12 adults and 27 juveniles represented as circles in autumn and 12 adults as triangles in spring). Significant predictors are those that the 95% confidence interval did not overlap with zero in the tested models. Orange (autumn) and green (spring) lines signal when the predictor was significant for that season, while grey lines show non-significant relationships. The response variable of average day to night flight ratio is not included in this figure because it is presented in the main text.


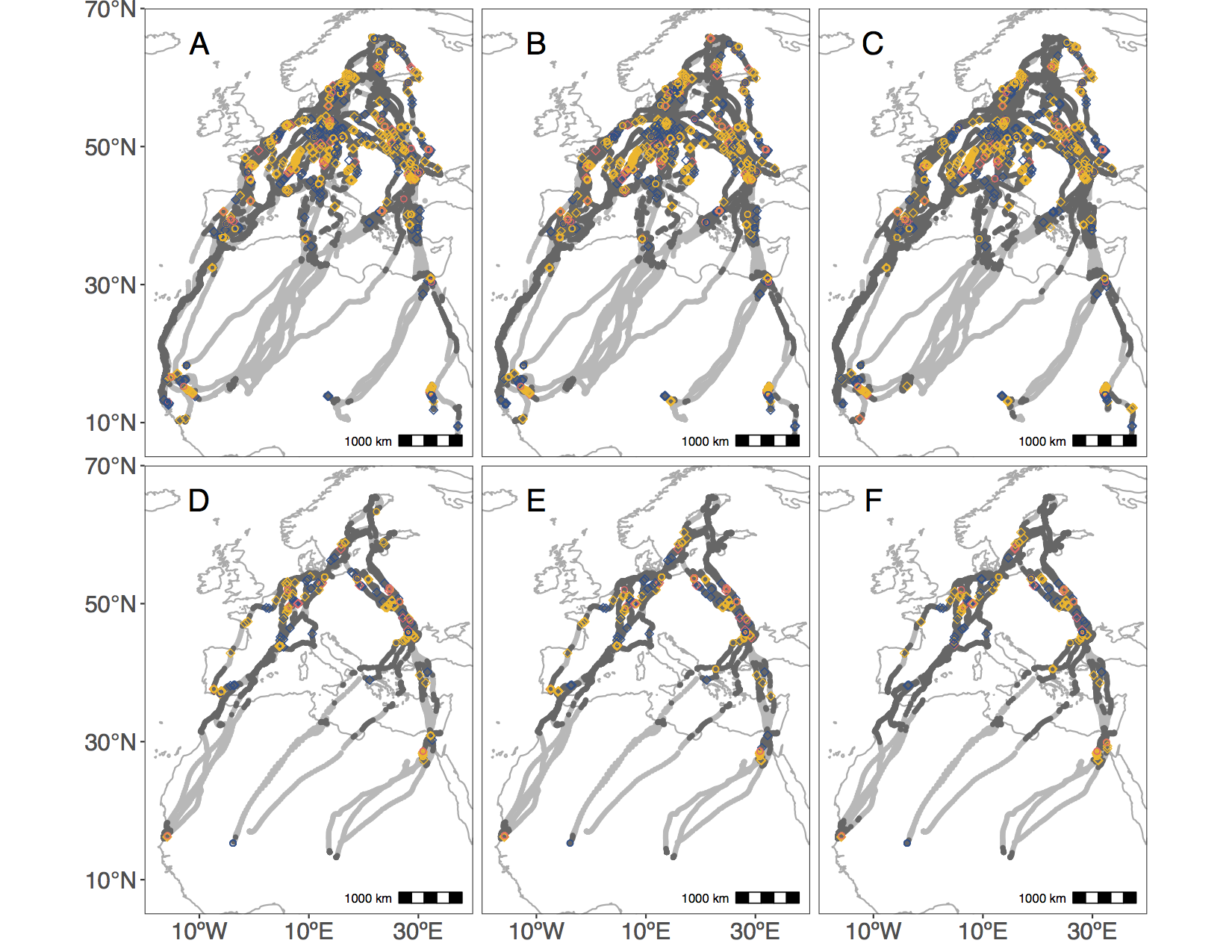


Figure S8.  Relocations with greater proportion of water bodies in a 5 km radius than expected by chance from random points sampled in the surroundings within a 35 (A and D), 50 (B and E) and 80 (C and F) km radius buffer, for autumn (A-C; 12 adults, 27 juveniles) and spring (D-F; 12 adults) migration of tracked Caspian terns. Relocations with greater proportion of water than expected by chance from the landscape within traveling segments during flight are shown as diamonds and in stationary periods as circles, and colored according to the time of day with dark blue for night, pink for dawn/dusk and yellow for daytime. Relocations that were not significantly different in proportion of water from what was expected from the landscape are shown in dark grey, and those with no variation in water (standard deviation had a value of zero, in areas such as the desert or over the sea) are shown in light grey.


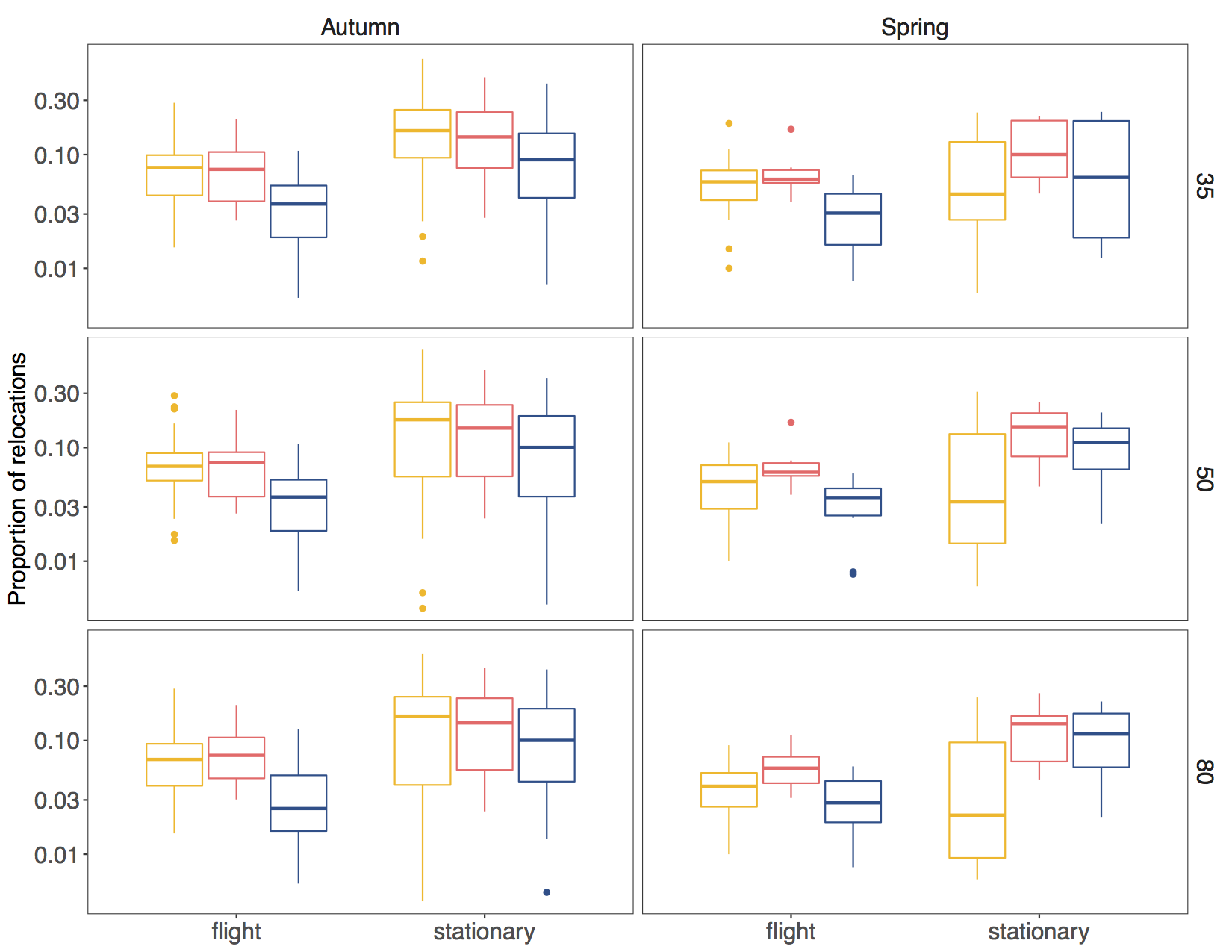
Figure S9. Proportion of relocations with significantly more available foraging habitat measured as proportion of overlap with water bodies, according to periods of migratory flight (ground speed > 10 km/h at both the start and end of 30-minute intervals) and stationary periods (ground speed < 10 km/h) within traveling days for tracked Caspian terns (27 juveniles in autumn and 12 adults in autumn and spring). Panel rows indicate 35, 50 and 80 km buffers used to compare observed overlap with water bodies in a 5 km radius to that offered by chance from the landscape. Color of boxplots indicates time of day, with yellow for daytime, pink for dawn/dusk and blue for nighttime. The middle thick line of the boxplots is the median, hinges of the box are the first and third quartiles, lines extend 1.5 of the inter-quartile range and outliers are represented by points. Note that the scale of the y-axis is logarithmic to aid visualization.


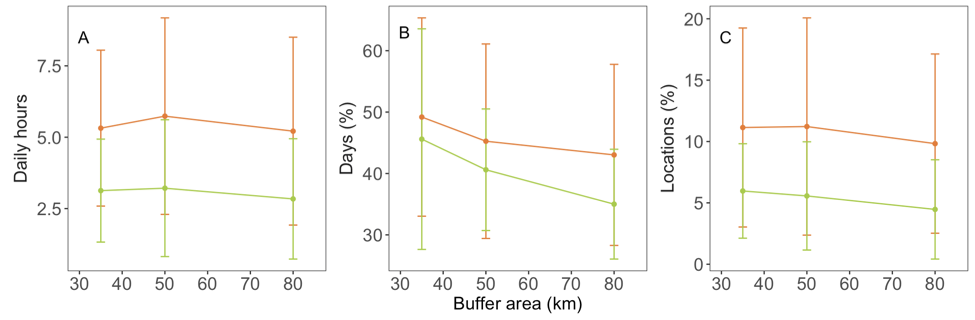


Figure S10. Average daily hours (A), percentage of days (B) and percentage of relocations (C) spent at locations with greater proportion of overlap with water than surrounding landscape, calculated by random points generated within 35, 50 or 80 km of observed points. Points show average values for all 39 tracked Caspian terns in autumn (orange, 12 adults and 27 juveniles) and spring (green, 12 adults). Error bars represent one standard deviation from average values. Relocations that had no possibility of habitat selection (over sea or desert, where variation in water overlap is 0) were excluded to calculate all three measures presented in the figure.
